# Schiff bases complexed with iron and their relation with the life cycle and infection by *Schistosoma mansoni*

**DOI:** 10.3389/fimmu.2022.1021768

**Published:** 2022-12-21

**Authors:** Juliana Virginio da Silva, Carla Cristina Moreira, Elisandra de Almeida Montija, Karina Alves Feitosa, Ricardo de Oliveira Correia, Nelson Luis de Campos Domingues, Edson Garcia Soares, Silmara Marques Allegretti, Ana Afonso, Fernanda de Freitas Anibal

**Affiliations:** ^1^ Departamento de Morfologia e Patologia (DMP), Laboratório de Inflamação e Doenças Infecciosas (LIDI), Universidade Federal de São Carlos (UFSCar), São Paulo, Brazil; ^2^ Laboratório de catálise orgânica e biocatálise, Universidade Federal da Grande Dourados, Dourados, Mato Grosso do Sul, Brazil; ^3^ Departamento de Patologia, Faculdade de Medicina de Ribeirão Preto, Universidade de São Paulo, Ribeirão Preto, São Paulo, Brazil; ^4^ Departamento De Biologia Animal, Instituto de BiologiaEstadual de Campinas, Universidade, Campinas, São Paulo, Brazil; ^5^ Global Health and Tropical Medicine (GHTM), Unidade de Parasitologia Médica, Instituto de Higiene e Medicina Tropical (IHMT), Universidade Nova de Lisboa (UNL), Lisboa, Portugal; ^6^ Instituto de Química de São Carlos (IQSC), Universidade de São Paulo (USP), São Paulo, Brazil; ^7^ Instituto Nacional de Investigação Agrária e Veterinária, I.P., (INIAV), Laboratório de Parasitologia, Oeiras, Portugal; ^8^ Laboratório de Parasitologia, Quantoom’s Bioscience, Nivelles, Bélgica, Belgium

**Keywords:** inflammation, BALB/c, *Biomphalaria glabrata*, *in vivo* – *in vitro*, schistosomicidal activity

## Abstract

**Introduction:**

The trematode *Schistosoma mansoni* causes schistosomiasis, and this parasite’s life cycle depends on the mollusk *Biomphalaria glabrata.* The most effective treatment for infected people is administering a single dose of Praziquantel. However, there are naturally resistant to treatment. This work has developed, considering this parasite’s complex life cycle.

**Methods:**

The synthetics compound were evaluated: i) during the infection of *B. glabrata*, ii) during the infection of BALB*/c* mice, and iii) during the treatment of mice infected with *S. mansoni.*

**Results and Discussion:**

For the first objective, snails infected with miracidia treated with compounds C1 and C3 at concentrations of 25% IC50 and 50% IC50, after 80 days of infection, released fewer cercariae than the infected group without treatment. For the second objective, compounds C1 and C3 did not show significant results in the infected group without treatment. For the third objective, the mice treated with C3 and C1 reduced the global and differential cell count. The results suggest that although the evaluated compounds do not present schistosomicidal properties when placed in cercariae suspension, they can stimulate an immune reaction in snails and decrease mice’s inflammatory response. In general, we can conclude that compound C1 and C3 has an anti-schistosomicidal effect both in the larval phase (miracidia) and in the adult form of the parasite.

## 1 Introduction

Schistosomiasis is a chronic parasitosis that affects tropical and subtropical areas of the globe. This disease is related to inadequate sanitation conditions; therefore, it is more reported in probing communities, and 779 million people are at risk of infection (1, 2). Trematodes of the Schistosoma genus cause it.

The trematode’s life cycle depends on the presence of an intermediate host: the snails of the genus *Biomphalaria*. Within them, larval development (miracidia to cercaria) occurs. The cercariae are released by snails and infect vertebrates (definitive host), where the parasite reaches its adult form. Within the body, the parasite reaches the liver and intestine. The individual affected by this disease may present with the most varied symptoms ranging from dermatitis and fever to an enlarged liver and spleen (3). Annually, schistosomiasis affects over 290 million individuals and causes 280,000 deaths (2, 4). For this reason, it is one of the world’s most significant neglected tropical diseases (1).

In Brazil, schistosomiasis is caused by *Schistosoma mansoni*, and *Biomphalaria glabrata* is the primary, intermediate host (4, 5). Endemic areas are Brazil’s Northeast and Southeast regions (4).

The control of schistosomiasis consists of two main methods: prevention and treatment (2). The first involves eliminating host snails and improving sanitation. Snail management is essential in reducing the number of intermediate hosts. However, this management is done with molluscicide compounds, which can result in environmental destruction (2). The second method is linked to the mass drug administration of the population living in risk areas. However, the only medication the World Health Organization (WHO) recommended for treating this helminthiasis is Praziquantel (6).

Although Praziquantel is the most widely used treatment for schistosomiasis worldwide, decreased sensitivity to the drug has already been observed in some strains (2, 6, 7). Within this context, the work carried out by Pinto-Almeida and collaborators (8) evaluated the tegument of resistant and susceptible strains to Praziquantel. This study showed that worms susceptible to Praziquantel exposed to the drug had more severe tegumentary damage than resistant worms, which showed only minor changes. In addition, unlike the susceptible strain, resistant worms were viable after exposure to Praziquantel and gradually recovered total motility after removing the drug. Another disadvantage of this drug is its ineffectiveness against the immature forms of the parasite. That is, Praziquantel is only effective against the adult parasite and, therefore, its most important use is in individuals already infected and not in preventing infection (7). These factors suggest the need for research to develop new schistosomicidal compounds.

In this scenario, searching for compounds with an active principle different from Praziquantel is essential for developing new treatments. The Schiff bases are a class of molecules that has been a recurrent research theme in both inorganic, organic, and biological fields. One of the premises of the potential of this compound is the similarity of structural properties with some biological systems (9).

The first record of the synthesis of Schiff’s Bases was in 1864 by the German chemist Hugo Schiff (1834–1915) (10). Schiff bases are condensation products of primary amines with carbonyl compounds (11). Due to the structural similarity of these compounds to biological systems, they form an important class of organic compounds most widely used in medicine (11). There are reports of using these compounds as antifungal (12) and antibacterial (13) agents. Within this context, many Schiff bases are known to be medicinally essential and are used to yield medicinal compounds.

Another relevant property of Schiff bases is their ability to form complexes with metals (14). In the last decades, much research works on the synthesis and pharmacology of Schiff’s Bases complexed with metals (15–17) since metal ions make the connection between drug substances and pathogenic organisms (18). Schiff’s Bases complexed with metals exhibit biological activities, such as antibacterial, antifungal, anticancer, antioxidant, anti-inflammatory, antimalarial, and antiviral activity (11, 18–20).

Although the Schiff Bases have wide application, they have been little described in the literature as potential anthelmintic agents (11, 21). For *S. mansoni*, the reports show that the Schiff Bases tested are not complexed with metals and were only tested in the adult form of the parasite (22, 23). There is then a gap with an exploratory potential: to evaluate the effect of Schiff Bases complexed with metals in the adult and larval form of *S. mansoni.*

The choice of metal is a fundamental step, as described by Coelho (24). Changing the metal changes the activity of the compound. The literature shows that metals can be used as anti-schistosomiasis agents (25, 26). In this context, the iron particles have demonstrated activities against helminths (*Toxocara vitulorum*) *in vitro* (25) and against *S. mansoni* adult worms *in vitro* (27).

Therefore, the present work sought to explore the effects of two Schiff Bases complexed with iron (Bis(2-((E)-((4-methoxyphenyl)imino)methyl)phenoxy)iron(III) chloride (C1) and Bis(2-((E)-((4-nitrophenyl)imino)methyl)phenoxy)iron(III) chloride (C3)) at three points in the *S. mansoni* cycle: during *Biomphalaria glabrata* infection with *S. mansoni* miracidia; during to *BALB/c* mice infection with *S. mansoni* cercariae; and after to *BALB/c* mice infection with *S. mansoni* cercariae.

## 2 Methodology

### 2.1 Compounds

Two compounds (C1 and C3) from the condensation reaction between salicylaldehyde and aromatic amines complexed with iron were tested. All concentrations used in this work (see **Table 1**) were based on the calculation of the inhibitory concentration of 50% viability (IC50) of the parasite *Leishmania infantum* in its promastigote form (24).

**Table 1:** Nomenclature and concentration of the compound used.

Compound	Nomenclature	IC_50_ (µg/mL)	25% IC_50_ (µg/mL)	50% IC_50_ (µg/mL)
C1	Bis(2-((E)-((4-methoxyphenyl)imino) methyl)phenoxy)iron(III) chloride	16,22	4,055	8,11
C3	Bis(2-((E)-((4-nitrophenyl)imino) methyl)phenoxy)iron(III) chloride + Fe	28,69	7,1725	14,345

### 2.2 Snail and parasite collection

Healthy *B. glabrata* snails were provided by Unicamp’s Helminth Laboratory, Campinas, São Paulo, Brazil. Snails were bred, according to (28), to increase the number of individuals.

The Sergipe (SE) strain of *S. mansoni* was used in all experiments. Miracidia from *S. mansoni* were obtained from clean eggs extracted from the livers of previously infected mice. The methodology was conducted following the standard operating protocols of Prof. Dr. Vanderlei Rodrigues from the Department of Biochemistry and Immunology at the Faculty of Medicine of Ribeirão Preto – USP. Hatched miracidia were observed under a microscope and counted.

*S. mansoni* cercariae were obtained from experimentally infected *B. glabrata* snails. They were placed individually in 24-well culture plates containing 1 ml of distilled water (dH2O), followed by exposure to artificial light for 1h30 (29). Hatched cercariae were observed under a microscope and counted.

### 2.3 BALB/c mice

The experimental design was established following the ethical principles of animal experimentation adopted by the Brazilian Society of Laboratory Animal Sciences and approved by the Ethics Committee on Animal Experimentation at the Federal University of São Carlos, São Paulo, Brazil (CEUA n. 009/2014).

Females of the *BALB/c* mice from the *vivarium* of the Faculty of Sciences Pharmaceuticals of Ribeirão Preto, São Paulo, Brazil, at four weeks of age, free of specific pathogens, were used in this work. The animals were kept in the *vivarium* of the Department of Morphology and Pathology of the Federal University of São Carlos, with food and water ad libitum. The temperature was maintained at 25 °C, and the luminosity was controlled with 12h of light and 12h of dark.

### 2.4 Drug effect during *B. glabrata* infection with *S. mansoni* miracidia

One hundred snails of the species *B. glabrata* were used. They were divided in five groups: (Inf) control; (C1_0,25_) miracidia treated with C1 at a concentration of 25% of the IC50 (4,05 µg/mL); (C1_0,50_) miracidia treated with C1 at a concentration of 50% of the IC50 (8,11 µg/mL); (C3_0,25_) miracidia treated with C3 at a concentration of 25% of the IC50 (7,17 µg/mL) and (C3_0,5_) miracidia treated with C3 at a concentration of 50% of the IC50 (14,34 µg/mL).

Culture plates with 24-well were prepared, respecting the particularities of each group. In each well, 15 miracidia were placed in a solution of C1 or C3 compounds at pre-defined concentrations. The culture plates with snails of the Inf group contained only distilled water and DMSO (3%) (solvent for compounds C1 and C3) and, therefore, was used as a control for this experiment.

Healthy *B. glabrata* snails were placed individually in 24-well culture plates and exposed to artificial light for 12h. At the end of the infection, the snails were kept in the dark environment and at a controlled temperature to prevent cercariae from being released at random (26).

At 30, 45, 60, and 80 days after parasite infection, snails from each group were placed individually in 24-well culture plates containing 1 mL of distilled water (dH2O), followed by exposure to artificial light for 1h30 (29). Shed cercariae were observed under a microscope and counted (26).

### 2.5 Drug effect during to *BALB/c* mice infection with *S. mansoni* cercariae

Twenty-five female mice of the *BALB/c* mice were used in this stage of the work. They were divided in five groups: normal cercariae (Inf); cercariae treated with C1 at a concentration of 25% of the IC_50_ (C1_0,25_); cercariae treated with C1 at a concentration of 50% of the IC_50_ (C1_0,5_); cercariae treated with C3 at a concentration of 25% of the IC_50_ (C3_0,25_); cercariae treated with C3 at a concentration of 50% of the IC_50_ (C3_0,5_).

*S. mansoni* cercariae were obtained from laboratory-bred infected *B. glabrata* snails (29). Hatched cercariae were observed under a microscope and viable cercariae were selected and counted. Eighty cercariae were placed in a test tube that contained a solution of C1 or C3 compounds at pre-defined concentrations. The test tubes for the Inf group contained only distilled water and DMSO (3%) (solvent for compounds C1 and C3) and, therefore, was used as a control for this experiment.

Infection by tail immersion (TI) was done according to Olivier and Stirewalt (30). Mice were immobilized without anesthetic. Moreover, their tails were exposed to approximately 2 hours of suspension, respecting the particularities of each group.

Forty-eight days after the parasite infection, feces were collected from the animals for the Kato Katz. Animals were euthanized using a CO2 chamber, and their livers were selected for histological analysis.

### 2.6 Drug effect after to *BALB/c* mice infection with *S. mansoni* cercariae

Fifteen females of the *BALB/c* mice were used in this stage of the work. Infection by tail immersion (TI) was done according to Olivier and Stirewalt (30). Mice were immobilized without anesthetic, and their tails were exposed for approximately 2 hours to suspension with cercariae. Each mouse was infected with 80 *S. mansoni* cercariae. After parasite infection, the animals were randomly divided into three groups: Control (Inf), Treated with C1 (C1), and treated with C3 (C3).

After 45 days of infection, eggs of *S. mansoni* were found in the feces of all mice. Then, treatment was started. Compounds C1 and C3 were administered *via* gavage, respecting the group, in 3 doses on consecutive days at a concentration of 40 mg/kg animal. The control group animals received only the compounds’ solvent (DMSO 3%).

The animals were euthanized using a CO2 chamber 12 days after the start of treatment, that is, 60 days after parasite infection. Livers were selected for histological analysis, and peripheral blood washed from the peritoneal cavity was collected for total and differential leukocyte count and immunoenzymatic assays.

The egg count was performed two moments:45 days after parasite infection, that is, before treatment (BT), and 60 days after parasite infection, that is, after treatment (AT), following the Kato-Katz technique (31).

### 2.7 Kato-Katz

The thick Kato-Katz smear (31) is the method the World Health Organization recommended to quantitatively evaluate *S. mansoni* eggs (4). The method was performed as described by Katz and collaborators (31).

Three slides from each sample were prepared. Each slide was screened by optical microscopy to identify and quantify *S. mansoni* eggs. The egg values per gram were calculated based on the average number of eggs counted in three slides and multiplied by 24 (4).

### 2.8 Histological analysis

The extracted livers were immediately fixed in formaldehyde 10% buffered. Specimens were routinely processed, embedded in paraffin blocks, and sectioned into sections of 5 μm (6). The slides were stained with Gomori’s Trichrome for examination under light microscopy. Each slide was screened by optical microscopy to identify fibrosis, granulomas, and inflammatory infiltrates. Slides were photographed using a Leica DMRX microscope with a camera.

### 2.9 Global and differential counts of cells

Peripheral blood samples were obtained through the left brachial vein. Absolute leukocyte counts were measured using a Neubauer chamber with Turk solution for 1:20 dilution (Gentian Violet 0.002 g, 3% Acetic acid). Peripheral blood was collected using EDTA as an anticoagulant. The absolute number of different leukocytes was calculated using differential counts on blood smears stained with Panoptic Rapid (LB, Laborclin).

Peritoneal cavity lavage (LPC) cells were collected after the injection of 3 ml PBS containing 0.5% sodium citrate. Counts for the total number of leukocytes in the peritoneal cavity were performed using a Neubauer chamber, and differential counts were obtained from slides prepared using cytospin (Serocito Mod. 2400 Fanen; 1000 rpm/3 min) and stained with Panoptic Rapid (LB, Laborclin).

### 2.10 Immunoenzymatic assays

Peripheral blood samples were obtained through the left brachial vein. Peripheral blood was collected using EDTA as an anticoagulant. IFN-γ and IL-4 cytokines in plasma were quantified by commercial ELISA sets (BDOptEIATM, Becton Dickinson, USA) according to the manufacturer’s instructions. Each assay was performed in duplicate.

### 2.11 Statistical analysis

Data were preliminarily verified by Kolmogorov–Smirnov test. Data sets were analyzed by one-way analysis of variance (ANOVA) followed by Dunnett’s test, and values with p ≤ 0.05 were considered significant. The statistical analysis was performed in GraphPad Prism 8.0.1 software.

## 3 Results

### 3.1 Drug effect during *Biomphalaria glabrata* infection with *S. mansoni* miracidia

At 30, 45, 60, and 80 days after parasite infection, snails from each group were exposed to artificial light for 1h30. Shed cercariae were observed under a microscope and counted. In the 30 and 45 days after parasite infection, free swimmers’ cercariae were not obverted in the watery environment. On the other hand, at 60 and 80 days after parasite infection, cercariae were observed. Results are shown in **Table 2**.

**Table 2:** Estimation of the number of *S. mansoni* cercariae shed of snail infected with treated (C10,25, C10,5, C30,25, C30,5) and untreated miracidia (Inf).

Time	Group	Cercarie (Mean ± SEM)	Reduction (%)
60	Inf	14,00 ± 2,31	–
	C1_0,25_	4,67 ± 0,67	66,66*
	C1_0,5_	8,00 ± 2,31	42,86
	C3_0,25_	8,67 ± 0,67	38,09
	C3_0,5_	19,33 ± 1,77	
80	Inf	87,67 ± 4,17	–
	C1_0,25_	10,00 ± 1,15	88,59***
	C1_0,5_	8,00 ± 2,31	90,91***
	C3_0,25_	9,33 ± 1,33	89,35***
	C3_0,5_	16,67 ± 2,67	80,98***

Data 60 days after parasite infection and 80 days after parasite infection. Data are expressed as the mean ± SEM of three independent experiments (*p ≤ 0.05 and ***p ≤ 0.0001 compared with Inf).

At 60 days after parasite infection, only snails the group C1_0,25_ showed a significant decrease in the number of cercariae eliminated compared to snails infected with untreated miracidia. In this case, the reduction was about 67% in the number of cercariae shed.

In contrast, 80 days after parasite infection, all groups showed a significant reduction in the number of cercariae shed. Snails of group C1 reduced 88% and 90% cercariae in the concentration of 25% and 50%, respectively, compared to the snails infected with untreated miracidia. Similarly, snails of group C3 reduced 89% and 81% cercariae in the concentration of 25% and 50%, respectively, compared to the snails infected with untreated miracidia.

A qualitative evaluation was carried out on the shed cercariae. Cercariae shed by the infected snails in the presence of C1 and C3 presented lethargy. In contrast, the cercariae eliminated by the snails of the Infected group without adding compost showed normal mobility.

### 3.2 Drug effect during to *BALB/c* mice infection with *S. mansoni* cercariae

**Figure 1:**
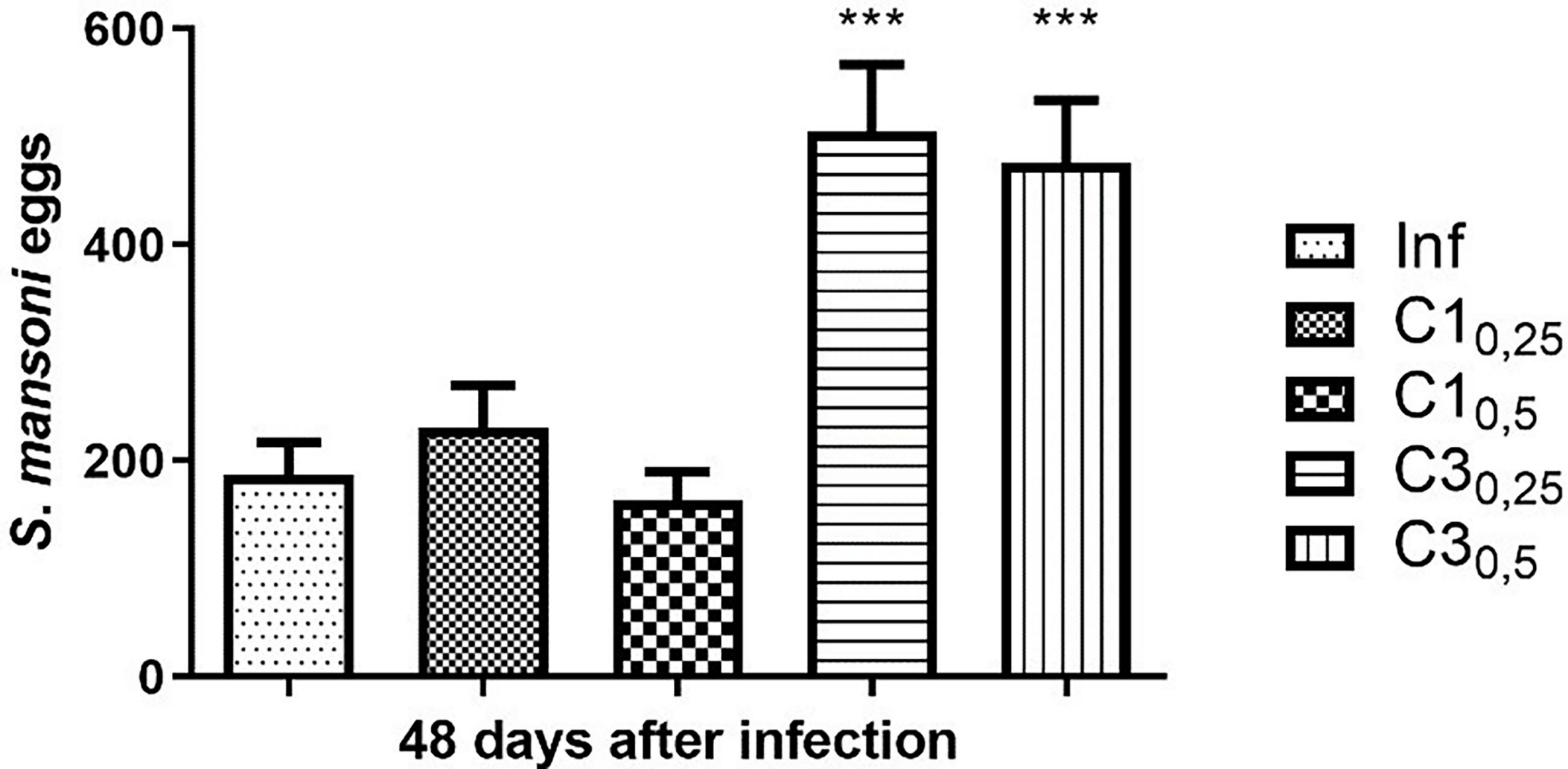
Estimation of the number of *S. mansoni* eggs in the faeces of mice infected with treated (C1_0,25_, C1_0,5,_ C3_0,25,_ C3_0,5_) and untreated cercariae (Inf). Data are expressed as the mean ± SD of three independent experiments (***p ≤ 0.0001) compared with Inf). Source: Author. Software: GraphPad Prism v8.

Forty-eight days after parasite infection by tail immersion, feces were collected from the animals for the Kato Katz. The results are shown in **Figure 1**.

Mice of group C1_0,25_ showed a 23% increase in the number of eggs compared to mice infected with untreated cercariae. In contrast, mice of group C1_0,50_ showed a decrease of 13% when compared to the positive control. Statistically, compound C1 at the two concentrations tested did not show a significant difference in the number of eggs compared to mice infected with untreated cercariae.

On the other hand, mice infected with cercariae treated with the compound C3 at concentrations 25% and 50% of the IC50 showed a significant increase (respectively 170% and 154%) in the number of eggs compared to mice infected with untreated cercariae.

**Figure 2:**
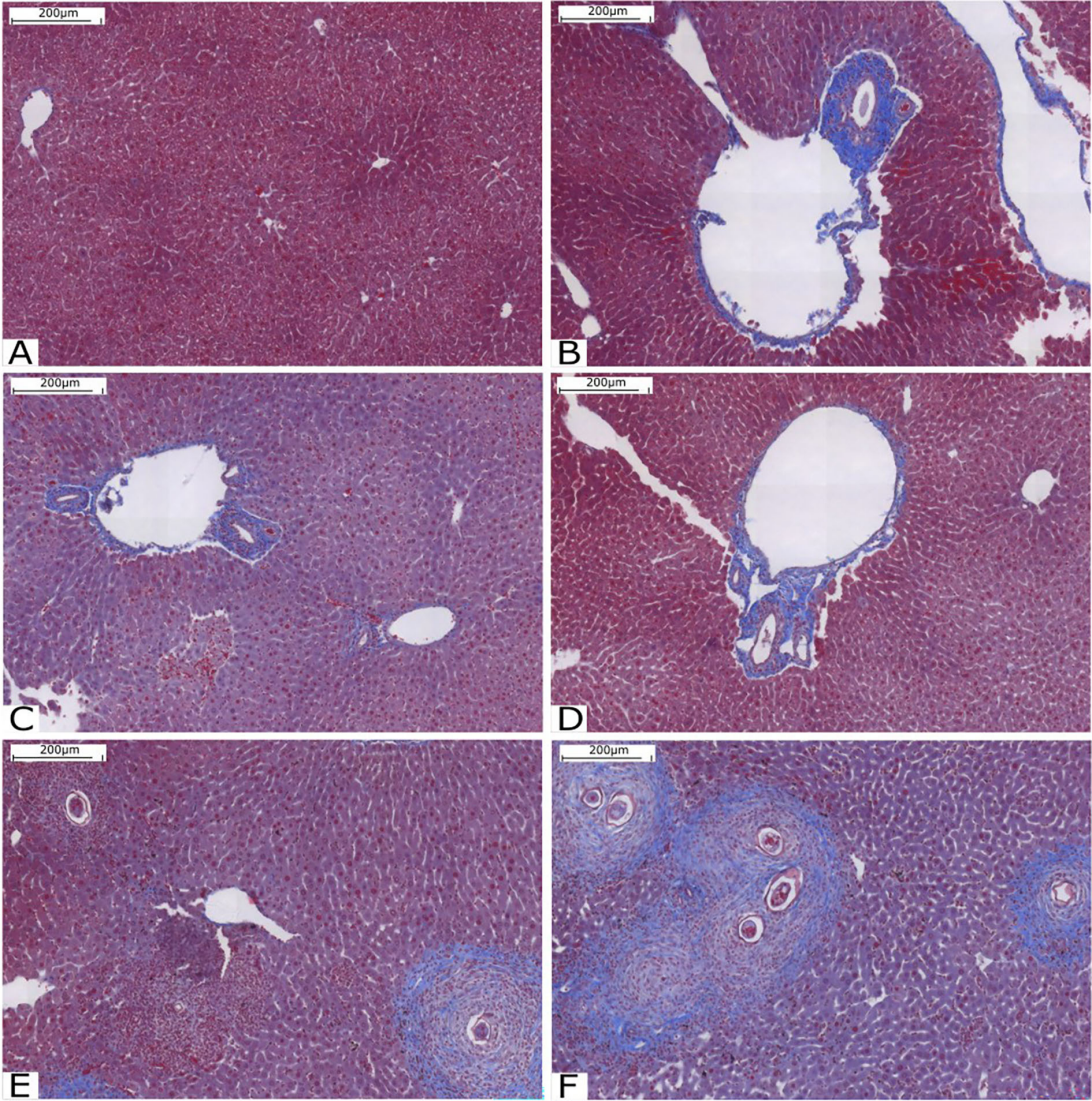
Histological analysis of the liver stained with Gomori’s trichrome. **(A)** Liver tissue without injury or inflammation; **(B)** Infected Group; **(C)** Group C1_0.25_; **(D)** Group C1_0.50_; **(E)** Group C3_0.25_ and **(F)** Group C3_0.50_. Source: Author. Software: PannoramicViewer.

Animals were euthanized using a CO_2_ chamber, and their livers were selected for histological analysis. Liver fibrosis was assessed by staining with Gomori trichrome, with collagen fibers stained in blue and hepatocytes stained in red. Qualitative results are shown in **Figure 2**. It is possible to observe that the pattern of fibrosis and lesion in the livers of animals infected with cercariae treated with compound C1 in the two concentrations is very similar to the pattern presented in the mice of the Infected group. However, the amount of collagen fiber was significantly increased in the group with mice infected with the compound C3. In these animals, fibrosis was found around the granulomas. Compared with the other groups, the groups treated with C3 exhibited increased collagen deposition in the portal tracts and within the granulomas.

### 3.3 Drug effect after to BALB/c mice infection with *S. mansoni* cercariae

**Figure 3:**
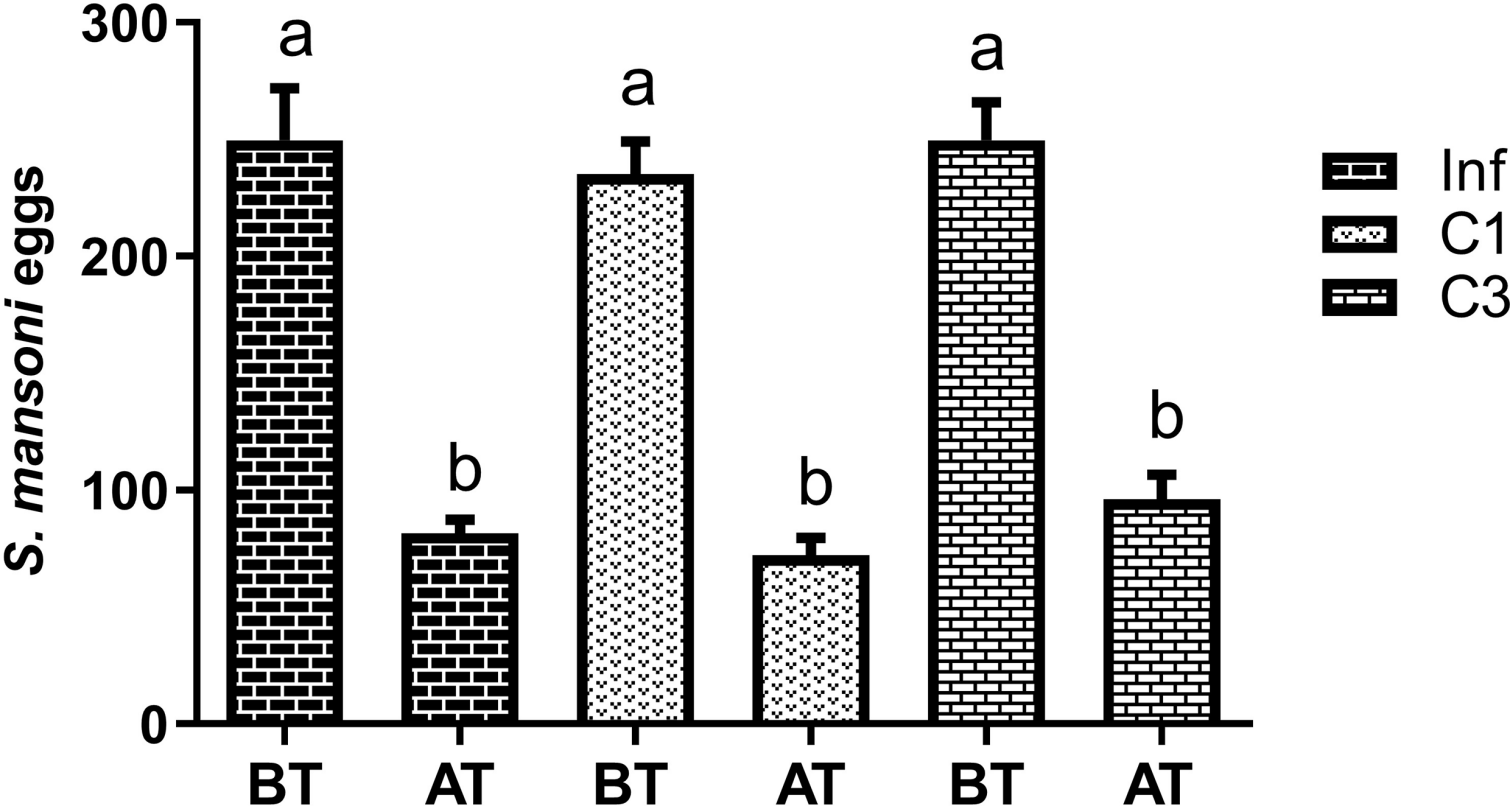
Estimation of the number of *S. mansoni* eggs in the faeces of mice infected untreated cercariae. BT means before treatment (45 days after parasite infection) and AT means after treatment (60 days after parasite infection). Data are expressed as the mean ± SD of three independent experiments. Differences were considered significant when p < 0.05. Groups with the same letters did not present a statistically significant difference. Source: Author. Software: GraphPad Prism v8.

At this stage, the egg count was performed in two moments: 45 days after parasite infection, that is, before treatment (BT), and 60 days after parasite infection, that is, after treatment (AT). The counting results are shown in **Figure 3**.

**Figure 3** shows that more eggs were in the animals’ feces 45 days after parasite infection (BT) than 60 days after (AT). Although this decrease was significant, it was statistically equal between the groups studied. Thus, the compounds C1 and C3 had no effect in reducing the number of eggs deposited in the animals’ feces.

**Figure 4:**
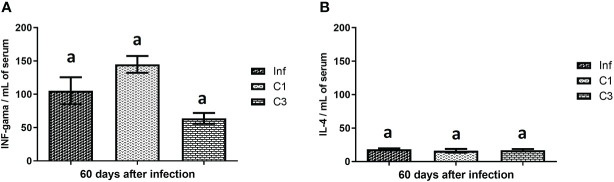
Cytokines levels on day 60 post parasite infection with *S. mansoni*. **(A)** INF-gama pg/mL; **(B)** IL-4 pg/mL. Data is presented as mean ± SD. Comparison are done between the Inf group. Differences were considered significant when *p* < 0.05. Groups with the same letters did not present a statistically significant difference. Source: Author. Software: GraphPad Prism v8.

We assessed the level of cytokine in the animals’ plasma on day 60 post-infection and post-treatment. These results are shown in **Figure 4**. It is possible to notice that the treated groups (C1 and C3) showed no difference in the levels of INF-gamma (**Figure 4**) and IL-4 (**Figure 4**) when compared to the control group infected without treatment.

**Figure 5:**
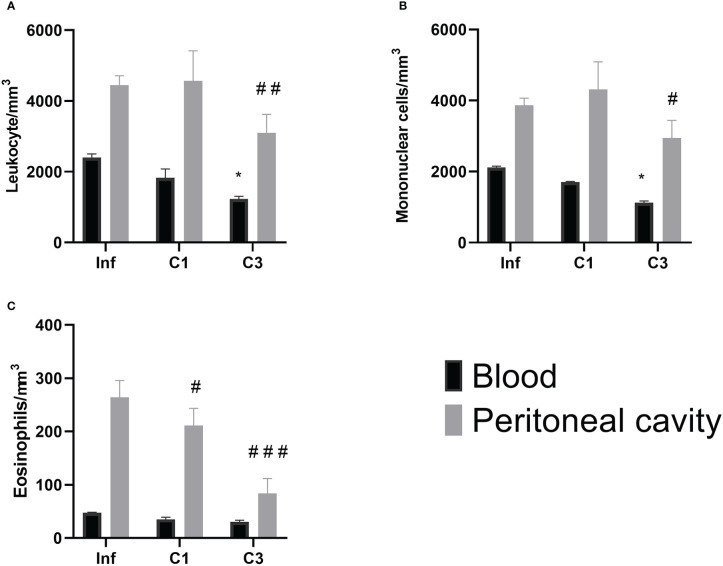
Global and differential counts of blood and peritoneal cavity lavage leukocytes on day 60 post parasite infection with *S. mansoni*. **(A)** Leukocytes/mm^3^; **(B)** Mononuclear/mm^3^; **(C)** Eosinophils/mm^3^. Data is presented as mean ± SD. Comparison are done between the Blood Infect group (*) and Peritoneal cavity lavage Infect group (^#^). (* or ^#^p ≤ 0.05, ^##^p ≤ 0.001 and ^###^p ≤ 0.0001). Source: Author. Software: GraphPad Prism v8.

However, when evaluating the global and differential cell counts, it is possible to notice a difference between the untreated infected group and those treated with C1 or C3. Our results showed that treatment with C3 reduced the number of global leukocytes compared to the untreated infected control (**Figure 5**) in the blood and the washing of the peritoneal cavity. A significant decrease was observed in mononuclear cells in the group treated with compound C3 compared to the infected group (**Figure 5**). Again, this reduction was observed both in the blood and in washing the peritoneal cavity.

About the number of eosinophils (**Figure 5**), there was a reduction in the number of these cells in the washing of the peritoneal cavity in both the group treated with C1 and C3 when compared to the infected group.

**Figure 6:**
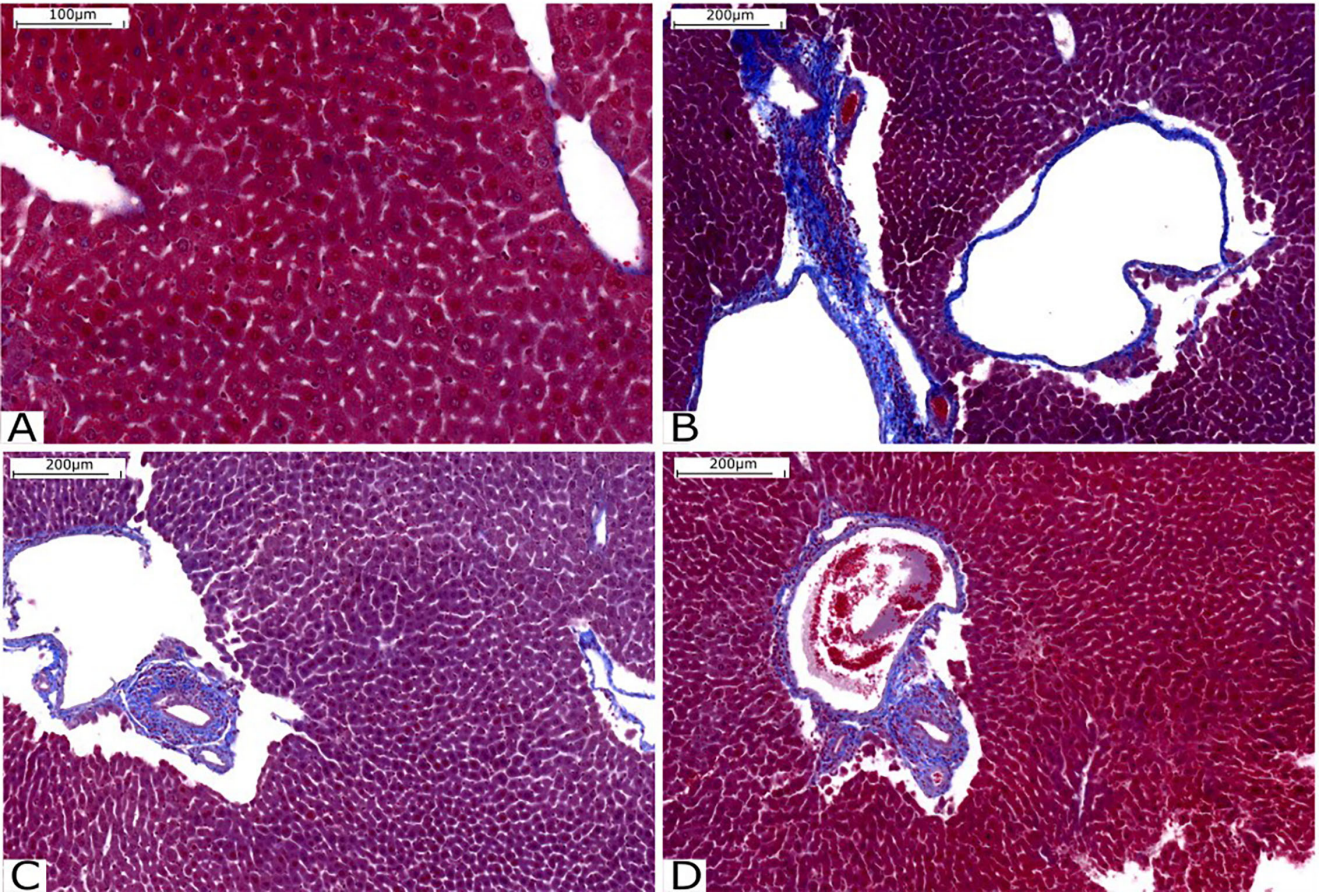
Histological analysis of the liver stained with Gomori’s trichrome. **(A)** Liver tissue without injury or fibrosis. **(B)** Infected Group; **(C)** Group treated with C1 and **(D)** Group treated with C3. Source: Author. Software: PannoramicViewer.

Liver fibrosis was assessed by staining with Gomori trichrome, with collagen fibers stained in blue and hepatocytes stained in red. It is possible to observe that there is intense perivascular fibrosis in the untreated Infected group (**Figure 6**). However, perivascular fibrosis is moderate in the groups treated with compounds C1 (**Figure 6**) and C3 (**Figure 6**). The patterns of fibrosis in the livers of animals treated with C1 and C3 are very similar.

## 4 Discussion

As a neglected disease, schistosomiasis research receives little investment. Thus, there is a need to search for new compounds that are effective against schistosomiasis (32, 33). Because of this and knowing that the only available drug has been presenting some cases of loss of sensitivity, the search for new therapies is urgent (34). However, some studies evaluate the efficiency of compounds in the adult form of *S. mansoni* (6, 35–38). Therefore, these studies may present inefficient compounds in treating acute schistosomiasis and low activity against *S. mansoni* in immature stages. These limitations indicate the need to study the efficiency of compounds in different forms of the parasite.

Our work sought to explore the effects of two Schiff Bases complexed with iron (Bis(2-((E)-((4-methoxyphenyl)imino)methyl)phenoxy)iron(III) chloride (C1) and Bis(2-((E)-((4-nitrophenyl)imino)methyl)phenoxy)iron(III) chloride (C3)) at three points in the *S. mansoni cycle*: during *B. glabrata* infection with *S. mansoni* miracidia; during to BALB/c mice infection with *S. mansoni* cercariae; and after to BALB/c mice infection with *S. mansoni* cercariae. It is worth mentioning that both compounds do not have a molluscicidal effect since the massive mortality of these animals can trigger ecological imbalances over a long time (2).

Our results indicate that the presented, analyzed compounds had significant effects on the parasite life cycle at two of the three evaluated points. Among the most significant results we would like to highlight: are a decrease in the number of cercariae shed by infected snails in the presence of compounds C1 and C3; a decrease in the number of eosinophils in the peritoneal cavity in animals treated orally, with compounds C1 and C3 and a decrease in the number of leukocytes, mononuclear cell and eosinophils in the blood and peritoneal cavity lavage in animals treated, orally, with C3 compounds.

Feces from any host infected with *S. mansoni* contains the parasite’s eggs, releasing miracidia, which penetrate and infect snails. The parasites transform and develop in these snails over a few weeks to become cercariae. In our work, regardless of the experimental group, snails release cercariae after 60 days of infection. These results corroborate with previous data from the literature that show that most snails eliminate cercariae from the seventh week onwards (39).

Pinto-Almeida and colleagues in 2016 (40) report that there are gaps in the understanding of *in situ* host-parasite snail interactions, mainly due to the small size of the parasites, their non-homogeneous distribution in the snails’ tissues, and the variability among individual snails in terms of resistance and susceptibility to infection. The initial interaction between a hemocyte and sporocysts may be strongly influenced by physicochemical properties (41, 42). This interaction is mediated by substances secreted by sporocysts and by the recognition of sporocyst tegument molecules, leading to encapsulation of the parasite and cellular activation, which result in the production of highly toxic metabolites of oxygen and nitrogen associated with the killing parasite (41).

Effect of the drug during infection by *B. glabrata* by *S. mansoni* miracidia indicates that snails infected in the presence of compounds C1 and C3 in the two concentrations tested released a lower number of cercariae compared to control 80 days after parasite infection. The reduction in the number of cercariae released was more significant than 80% in all groups evaluated. In contrast, 60 days after parasite infection, only C1 in the IC50 concentration decreased the number (84%) of cercariae shed. It is worth mentioning that the cercariae shed by the infected snails in the presence of C1 and C3 showed lethargy and malformation, while the cercariae shed by the snails of the Infected group without the addition of compost were viable and had normal mobility and morphology.

Our results suggest that the compounds C1 and C3, in the two concentrations tested, may have promoted changes in the structure of miracidia, causing a more intense amoebocyte reaction around the sporocysts leading to their death. Similar to our results, the work carried out by Moustafa and colleagues (26) indicates that snails infected with miracidia treated with metal ions developed cercariae with abnormal morphology. In the same sense, Hahn and colleagues (43) work highlights the importance of nitric oxide as it is involved in the toxicity mediated by *B. glabrata* hemocytes against *S. mansoni* sporocyst.

Cercariae shed by the infected snails swim freely in the freshwater. When these larvae find the intact skin of the definitive host (mammal), infection with *S. mansoni* begins. In the process, cercariae actively penetrate the skin and lose their tails. Shortly after penetration, the cercariae become schistosomules. That is, they lose the glycocalyx present in their bodies, and there are changes in the load on the parasite’s tegument (44, 45). These physical and chemical modulations are associated with the first stage of the life cycle of *S. mansoni* in the definitive host.

The number of cercariae penetrating through the skin is not directly related to the degree of infection caused by the parasite. Smithers and Terry (46) show that, in mice, the conversion rate of cercariae to adult worms is about 40%. Only 40% of the larvae managed to complete the cycle and transform into adult worms. The low transformation rate is related to the host’s immune process at the penetration time (47). Samuelson and Caulfield (48) show that the cercarial glycocalyx can activate the host’s immune system at the penetration time. Thus, glycocalyx found on the surface of cercariae is antigenic (49). As schistosomules have fewer glycocalyx than cercariae, they are less susceptible to death than cercariae.

Thus, we can infer that the host’s immune response begins with recognizing glycocalyx antigens. Within this scenario, many factors have not yet been explored (47), but some studies report that when there is a prolongation in the exposure time of these antigens, the immune response is increased and sustained (50, 51). Moreover, the opposite is also true, and minor interactions lead to delayed or uninduced responses (52). In general, the invasion of *S. mansoni* induces inflammation and increased migration of cells presenting innate antigens, such as macrophages and dendritic cells, to the site of infection.

Our results show that the feces of animals infected with cercariae treated with compound C3 in the two concentrations tested had more eggs than the feces of animals in the control group. This result suggests that the C3 compound may have been able to cause integumentary changes in the cercariae that culminated in the camouflage of the schistosomula, possibly generating a delayed immune response. Therefore, this compound has no positive effects on the elimination of the parasite.

The compounds used in our work are complex with Fe. The literature indicates that metal ions prevented the occurrence of infection when cercariae were treated before the infection by either the tail immersion route (26). In our study, cercariae were treated during infection. For this reason, we hypothesize that compound C3 may have induced some integumentary change in cercariae, such as loss of glycocalyx. This would not prevent the penetration of the cercariae, but would indicate a lower immune response from the host. Future work may study the microscopy of cercariae and confirm this hypothesis.

In our work, the mice’s tails were in contact with the compounds tested for 2 hours. It is known that the process of penetration of cercariae in the skin can last up to 15 minutes (47, 53). Therefore, the first immunological responses occurred in the presence of the studied compounds. Incani and McLaren (54) show that 3 hours after parasite infection, it is already possible to notice neutrophils, mononuclear cells, and fibroblasts present in the region of the infection.

C3 compound is a p-nitrosalicilidenoanilina complex with Fe. That is, there is a nitro- Schiff base complex to Fe. Roriz and colleagues (55) report that nitro-Schiff bases are possible anti-inflammatory agents, reducing the migration of neutrophils recruited to the site. Within this scenario, compound C3 may have acted topically at the site of infection, and it may have contributed to a decrease in the migration of defense cells to the site of infection. This may have resulted in an immunological delay that favored the action of the parasite and the maintenance of the life cycle of *S. mansoni.*

As it was not possible to count the adult worms, we concluded that compound C3 intensified the parasitosis, assuming that there was no reduction in schistosome fecundity. This conclusion takes into account the effect of metal ions and the effect of the anti-inflammatory property of compound C3. The results observed in the histopathological analysis (Figs. 2E and 2F) indicate an increase in collagen deposits and fibrosis of granulomas in the livers of animals infected by cercariae treated with compound C3. Together, these animals had a higher number of eggs in their feces 48 days after infection.

The literature indicates that the egg is the main pathogenic factor in schistosomiasis, as it acts as an initiator of granuloma formation and is essential for the pattern of events that lead to the pathology associated with chronic infection (56, 57). As the granuloma develops, fibrosis and collagen are deposited from the activated cells and remain permanently in the tissue (58).

On the other hand, when we looked at the effects of compounds C1 and C3 administered orally to mice, we noticed a slight decrease in fibrosis when we compared the treated groups with the control. In this group, adult worms were also not counted. Therefore, the conclusions found were made assuming that there was no difference in the fecundity of the animals.

In our study, the fecal egg count of the mice in our third study group shows that the number of eggs at 45 days after parasite infection was close to the peak, as about 250 eggs were obtained in all groups. A new count was performed 60 days after the parasite infection. Here, there was a significant reduction in the number of eggs. However, as the reduction was statistically similar among all groups, we believe the decrease is related to the parasite’s life cycle and not the treatments performed.

Zanotti-magalhães and colleagues (59) estimated *S. mansoni eggs* in one gram of feces of mice infected with cercariae from *B. glabrata* 56 days after parasite infection. The results show that they found about 80 eggs per gram of mouse feces. These results corroborate those found in our study since our study average was 83. Therefore, we cannot affirm that the compounds C1 and C3, administered orally in previously infected mice, have an anti-schistosomicidal effect.

The immune response usually occurs against *Schistosoma* eggs and granulomatous inflammation around the deposited eggs. The soluble egg antigen stimulates this process, and this antigen stimulates a Th2 immune response that leads to the production of IL-4 and levels of eosinophils (60). The low number of eggs can imply a smaller amount of the soluble egg antigen and, consequently, a low amount of IL-4 (Th2).

The Th2 response reaches the apex concomitantly with the egg release apex, approximately 6 to 8 weeks after parasite infection. In mice, these Th2 profile cytokines play an essential role in forming granuloma, in the presence of eosinophils, and developing fibrosis (61). The infection progresses to a more chronic phase and regulation of this Th2 profile occurs along with a reduction in the size of the hepatic granuloma. This immunomodulation regulates the balance of Th1/Th2 responses, being triggered by IL-10.

The immunological results presented in this work show that IFN-gamma is more expressed than IL-4. This result, added to the number of eggs, indicates that the mice were slightly affected by the disease. Although our work has not done the quantification of IL-10, it is believed that immunomodulatory regulating the balance of Th1/Th2 responses is occurring. These conclusions are in line with the work of De Jesus and collaborators (61). They show that schistosomiasis of degree I has higher IFN-gamma values compared to the expression values of Th2 cells. Also, we can suggest that the Th2 response was out of balance with the initial chronic phase in our study model.

In the mice, Th2 responses contribute to granuloma formation and the presence of eosinophils in these lesions (61). The defense cell migration is typically found in the bloodstream to the site of the injury caused by the egg. The results obtained in our study point to this migration because the number of cells found in the peritoneal cavity was higher than the number found in the blood. Although in our study, the IL-4 values do not show a statistical difference in the study groups, it is notable that the number of recruited cells showed a difference.

In the liver, a cytokine-independent event is the mechanism of oxidizing agents (62). Schiff bases have already been described as potential antioxidant agents (63). Therefore, they may suggest that the antioxidant activity of the Schiff bases may contribute to the potential antifibrogenic action, which would trigger a smaller deposit of malefic collagen.

The main organs affected during *S. mansoni* infection are the liver, kidneys and spleen. In them, a pro-oxidant process occurs due to increased activity of eosinophil peroxidase and imbalance in antioxidant defense mechanisms (64). During *S. mansoni* infection, oxidative processes occur at the site of granulomatous inflammation and, on the other hand, the antioxidant capacity of the liver is reduced, leading to the generation of lipid peroxides (65). In this sense, the use of antioxidant compounds can increase the antioxidant action of the liver and positively modulate the improvement of fibrosis.

A work published by Malta and collaborators shows that when hepatic and pulmonary granulomas are smaller, eosinophils show a decrease in peroxidase activity (66). Although our work did not analyze any antioxidant markers, it is notable that both compounds C1 and C3 had a decrease in eosinophils found in the peritoneal cavity lavage. This result could indicate that the compounds are potential antioxidants and this would be positively modulating the antioxidant action of the liver and, consequently, contributing to the decrease in the degree of fibrosis when we compare infected animals without any type of oral treatment.

The oral treatment with compounds C1 and C3 at a concentration of 40 mg/Kg of mouse body weight shows a decrease in the fibrosis response, which can be promising for reducing damage caused by schistosomiasis in infected individuals.

In conclusion, this work sought to evaluate the efficiency of two Schiff bases complexed with iron as an anthelmintic agent in the three stages of the *S. mansoni* life cycle: during infection in *B. glabrata*; during the infection in mice and the treatment of mice infected with *S. mansoni*.

In general, we can conclude that compound C1 and C3 have an anti-schistosomicidal effect both in the larval phase (miracidia) and in the adult form of the parasite.

The results found throughout our work open the possibility for further studies that seek to understand the mechanism of action of compounds C1 and C3 both in the larval phase (miracidia) and in the adult form of *S. mansoni*. This work also paves the way for other Schiff bases to be tested as an anthelmintic agent.

## Data availability statement

The original contributions presented in the study are included in the article/supplementary material. Further inquiries can be directed to the corresponding author.

## Ethics statement

The animal study was reviewed and approved by Ethics Committee on Animal Experimentation at Federal University of São Carlos, São Paulo, Brazil (CEUA n. 009/2014).

## Author contributions

JS: primary author of the work. She participated in all the techniques and analyses of this work. She made graphs, tables, and figures and participated in writing the article. CM, EM, KF, and RC actively participated in the developed techniques. They participated in the analysis and discussion of the results of this step and in writing the article. ND: availability of the compounds used in this work. ES: participation active in histological techniques. He participated in the discussion of the results of this step. SA: availability of the necessary equipment for the technique of parasite infection. She participated in the analysis and discussion of the results of this step. AA: she participated in the analysis, discussion, and formulation of the results and the article’s writing. FA: primary advisor of the project. She actively participated in the work, and the techniques developed. She participated in the analysis, discussion, and formulation of the results and the article’s writing. All authors contributed to the article and approved the submitted version.
